# First evidence of *Klebsiella pneumoniae* infection in Aceh cattle: Pathomorphology and antigenic distribution in the lungs

**DOI:** 10.14202/vetworld.2021.1007-1013

**Published:** 2021-04-27

**Authors:** Darniati Darniati, Surachmi Setiyaningsih, Dewi Ratih Agungpriyono, Ekowati Handharyani

**Affiliations:** 1 Animal Biomedical Sciences, Department of Veterinary Clinic Reproduction and Pathology, Faculty of Veterinary Medicine, Graduate School, IPB University, Bogor, Indonesia; 2 Department of Animal Disease and Public Health, Faculty of Veterinary Medicine, IPB University, Bogor, Indonesia; 3 Department of Veterinary Clinic Reproduction and Pathology, Faculty of Veterinary Medicine, IPB University, Bogor, Indonesia

**Keywords:** Aceh cattle, capsular serotyping, histopathology, immunohistochemistry, *Klebsiella pneumoniae*, lung

## Abstract

**Background and Aim:**

*Klebsiella pneumoniae* is an emerging zoonotic and foodborne pathogen worldwide. Hypervirulent *K. pneumoniae* (hvKp) was reported as the causative agent of bovine mastitis. This is the first study in Indonesia that has been conducted to determine the capsular serotype of *K. pneumoniae*, pulmonary gross pathology and histopathology, and distribution of hvKp in the lungs of Aceh cattle.

**Materials and Methods:**

The presence of *K. pneumoniae* in Aceh cattle was investigated in two slaughterhouses in Banda Aceh and Aceh Besar, Indonesia. Lung tissues with gross pathological lesions were collected from 15 cattle presenting with depression, dehydration, or cachexia. The confirmation and capsular serotyping of *K. pneumoniae* isolates were performed using polymerase chain reaction. The tissues were stained with hematoxylin-eosin and immunohistochemistry to observe the histopathological lesions and the distribution of the hvKp antigens.

**Results:**

The pneumonic lesions identified in the lungs of Aceh cattle included hyperemia, hemorrhage, consolidation, and atelectasis. *K. pneumoniae* was isolated in all 15 lung tissues with pathological pneumonic lesions. Two patterns of infection were observed histopathologically. Acute infection was characterized by hyperemia, inflammatory cell infiltration, hemorrhage, bronchiolar epithelium hyperplasia, bronchial and bronchiolar obstruction with purulent exudates, edema, and atelectasis. On the other hand, chronic infection was defined by macrophage infiltration, emphysema, bronchial dilatation, pleural fibrosis, and alveolar wall thickening by interstitial fibrosis. Immunohistochemical staining using monospecific antisera induced by the hvKp isolate confirmed the presence of *K. pneumoniae*-specific antigens in the acute infection, predominantly in the bronchiolar, vascular, and alveolar areas. In contrast, generally diffuse infiltrates were found in the pleura and interstitial alveolar areas in chronic infection.

**Conclusion:**

hvKp can be detected in the lungs of Aceh cattle, representing acute and chronic infections. The distribution of *Klebsiella* antigens in the lung tissue was consistent with the histopathological findings.

## Introduction

*Klebsiella pneumoniae* is a facultative anaerobic, Gram-negative, rod-shaped, non-motile encapsulated bacterium capable of fermenting glucose and lactose, which can cause infection in humans, animals, and plants [1,2]. *K. pneumoniae* were classified into classical *K. pneumoniae* and hypervirulent *K. pneumonia* (hvKp) strains based on their genotypic and phenotypic biomarkers [3]. hvKp phenotypes are associated with the production of a thick polysaccharide capsule that contributes to the increased virulence and pathogenicity. The capsule is considered the most important virulence factor in *K*. *pneumoniae*, which is required to maintain survival in the environment and within the host [4,5]. The polysaccharide capsule is also important because it favors the attachment to the cell surface and the formation of biofilms [6-9]. In addition, lipopolysaccharides (LPSs) mediate the resistance to complement-mediated and neutrophil-mediated bactericidal activity, as well as the resistance to multiple antibiotics activity [9,10].

*K. pneumoniae* is considered one of the most important pathogens in public health because they are animal-borne bacterial pathogens (zoonoses) [1]. In humans, *K. pneumoniae* has been associated with pneumonia, urinary tract infections, bacteremia, and liver abscess, primarily in immunocompromised hosts [11,12]. However, hypervirulent strains have been reported to cause various severe infections in immunocompetent and young healthy individuals [13]. hvKp has been implicated in causing pulmonary lesions in animals, such as suppurative bronchopneumonia in sheep [14], purulent bronchopneumonia in sea lions [15,16], pneumonia in Boer goats [17], and pneumonia and bacteremia in non-human primates [18]. On the other hand, hvKp infection in cattle is widely recognized as the causative agent of mastitis [19]. However, there was little evidence of *K. pneumoniae* association with bovine respiratory disease. *K. pneumoniae* was isolated from 25% out of 150 pneumonic lungs of cattle in a slaughterhouse in Nigeria [20]. To the best of our knowledge, one study published the isolation of KP in only one pneumonic lung of Bali cattle in Indonesia [21], but the capsular serotypes, histopathology, and distribution of the bacteria are not available in the literature. Recently, 12 isolates belonging to hvKp have been detected from cattle nasal swabs in Southwest China [1]. However, the information about the capsular serotypes of *K. pneumoniae* in cattle is still limited, and the pathological evidence has been poorly investigated.

Information regarding *K. pneumoniae* infection in cattle respiratory system is insufficient; therefore, a study related to this topic is important to recognize the prevalence and pathogenesis of infection in cattle. This preliminary study aimed to determine the capsular serotype of K. pneumoniae, pulmonary gross pathology and histopathology, and distribution of hvKp in pneumonic lungs of Aceh cattle.

## Materials and Methods

### Ethical approval

This research has been approved by the Animal Care and Use Committee of Research and Community Services Institution, IPB University, Bogor, with approval number: 144/KEH/SKE/VII/2019.

### Study period and location

The study was conducted from August 2019 to April 2020 at the Microbiology and Pathology Laboratory, Faculty of Veterinary Medicine, IPB University, Bogor, Indonesia.

### Animal and sample collection

Pneumonic lungs of 15 Aceh cattle from two slaughterhouses with any signs of depression, dehydration, and cachexia were included in this study. The samples were collected immediately after slaughter and evisceration. The lungs with gross lesions such as hyperemia, hemorrhage, consolidation, and atelectasis were collected aseptically. The specimens were cut into two pieces; one cut was dipped into brain heart infusion broth (BHIB) for microbiology examination, while the other was fixed into 10% neutral formalin buffer. Lung specimens in BHIB were kept at −20°C for microbiological examination.

### Bacterial isolation

The microbiological examination was carried out at the Microbiology Laboratory of the Faculty of Veterinary Medicine, IPB University, Bogor. Lung specimens were enriched overnight in BHIB at 37°C for 48 h, followed by streaking onto MacConkey agar and blood agar plates. The inoculated plates were anaerobically incubated in a lighted candle jar to reduced aerobiosis. Every single colony was identified by colony morphology, string test, Gram staining, capsule staining, carbohydrate fermentation (sucrose, mannitol, lactose, and glucose), and biochemical tests (indole, citrate, TSIA, catalase, and urease).

### Polymerase chain reaction (PCR)-based identification

Identification of suspected colonies was performed by *rpoB* (RNA polymerase *β* subunit) PCR amplification specific for *K. pneumoniae*. Nucleic acids were extracted from each colony using the boiling method. Briefly, one colony was resuspended in 50 mL nuclease-free water, heated at 100°C for 10 min, and the supernatant was collected by centrifugation at 12.000 *g* for 5 min. The *rpoB* PCR was performed according to Chander *et al*. [22] to detect 108 bp using a primer pair of F-CAACGGTGTGGTTACTGACG and R-TCTACGAAGTGGCCGTTTTC. Amplification was performed in *Bio*-*Rad thermal cycler* (*Bio*-*Rad* Laboratories, Inc., Hercules, CA, USA) using the following amplification conditions: 95°C for 3 min; 30 cycles of 95°C for 30 s, 55°C for 90 s, and 72°C for 90 s; and a final step at 72°C for 10 min. The PCR products were separated in 1.5% agarose gel electrophoresis for 30 min at 120 V and visualized using UV transilluminator.

### Histopathology

Histopathological examination was carried out at the Pathology Laboratory of the Faculty of Veterinary Medicine, IPB University, Bogor. The histopathological examination procedure followed a method as described by Zhou and Moore [23]. The fixed lung specimens were processed overnight for dehydration, clearing, and impregnation using an automatic tissue processor. The tissues were embedded in paraffin blocks using an embedding station and serial sections of 4-5 μm thickness were cut using a microtome. Tissue sections were stained with hematoxylin-eosin. The histopathological lesions assessment was done descriptively according to the presence of pneumonia signs. Acute bacterial infection was characterized by the presence of inflammatory cell infiltration, hyperemia, edema, hemorrhage, and atelectasis. Meanwhile, the chronic infection was determined by the existence of emphysema, bronchiole dilatation, and fibrosis. The severity of the lesion was graded with the following criteria: Focal lesion distribution (low severity), multifocal (moderate severity), and diffuse (high severity).

### Immunohistochemistry (IHC)

Monospecific antisera produced by immunizing rabbits using hvKp isolates followed by absorption against non-hvKp were used. The tissue section that has been attached to a microscopic slide using poly-L-lysine 1% was deparaffinized, rehydrated with distilled water, and put under flowing with tap water for 5 min, followed with phosphate-buffered saline (PBS) Tween. The method followed the protocol that was supplied with the mouse and rabbit specific HRP/DAB (ABC) detection IHC kit (ab64264, Abcam, Cambridge, UK). Blocking endogenous activity was performed with immersion in 3% hydrogen peroxide at room temperature (20-25°C) for 15 min, followed by three washes in PBS Tween. Non-specific protein binding was blocked using 10% normal goat serum at 20-25°C for 30 min and washed with PBS Tween. Tissue sections were incubated overnight at 4°C with rabbit anti-*K. pneumoniae* polyclonal antibodies and followed by incubation with secondary antibody Dako REAL™ envision™/HRP, rabbit/mouse (ENV) for 30 min. The streptavidin-HRP was applied for 30 min at 20-25°C, and the section was visualized using Dako REAL™ DAB+chromogen in Dako REAL™ substrate buffer. The section was counterstained with Mayer’s hematoxylin and mounted with Aquamount.

## Results

### Bacterial isolation and identification

hvKp was isolated in all 15 pneumonic lungs. *K. pneumoniae* were characterized as non-hemolytic gray-white mucoid colonies on blood agar and small pink mucoid (lactose fermenter) colonies on MacConkey agar. The bacterial isolates were rod-shaped and pink color in the Gram staining test. Biochemical tests showed negative results for indole, motility, and methyl red tests; and positive results for citrate, Voges-Proskauer, TSIA (A/A), and sugar fermentation tests. The hypermucoviscosity phenotype was defined by the string test, in which the formation of viscous strings greater than 5 mm in length was observed on spreading with an inoculation loop. The species identity was confirmed by the presence of 108 bp band of the *rpoB* region.

### Gross pathological lesion

**Figure-1:**
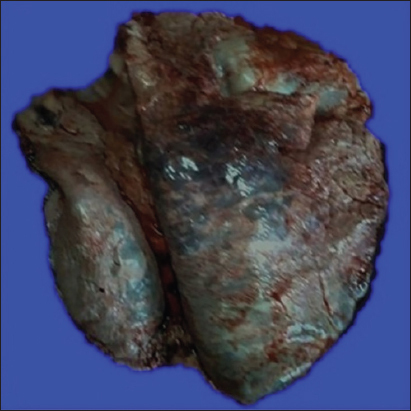
Dark red discolored area in acute form.

**Figure-2:**
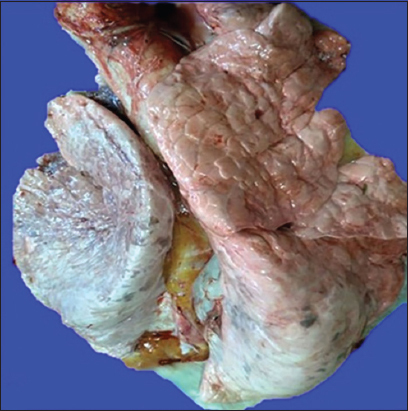
Gray-pink appearance in chronic form.

The main lung lesions observed in most of the cases were pneumonic patches with hyperemia, hemorrhages, atelectasis, and consolidation of the left and right ventral lobes with dark red-discolored areas to gray-pink appearance (Figures-1 and 2). Emphysema was detected in some of the lung tissue samples.

### Histopathology

**Figure-3:**
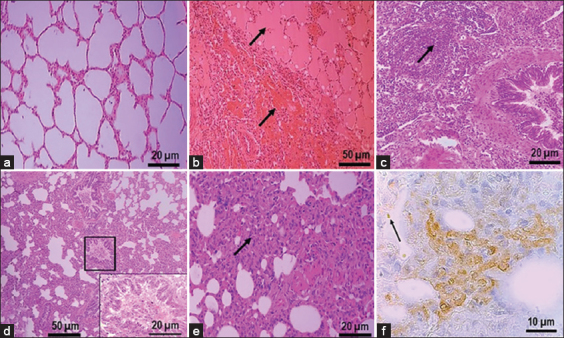
Lung histopathological lesions with suggested in acute infection. (a) Normal alveoli. (b) Lung edema and hemorrhage. (c) Inflammatory cell infiltration within the alveoli. (d) Bronchiolar epithelial hyperplasia (box) and obstruction with purulent exudate. (e) Atelectasis. (f) *Klebsiella pneumoniae* within macrophages of alveolar wall. *K pneumoniae* with intact capsule in areas less affected by inflammation (arrow).

The general tissue responses to hvKp were observed in all 15 lung samples, which could be attributed to acute and chronic pneumonia with purulent bronchopneumonia and interstitial bronchopneumonia. Acute inflammatory responses were characterized by the presence of hyperemia (congestion), edema, and hemorrhage within intra-alveolar spaces (Figure-3b), and inflammatory cells infiltration in the alveolar spaces (Figure-3c). The alveolar lumen of pneumonic lungs showed narrowing compared with normal lungs (Figure-3a). In addition, bronchiolar epithelium hyperplasia, peribronchiolar infiltration with inflammatory cells, bronchial and bronchiole obstruction with purulent exudate (Figure-3d), and collapse of lung or atelectasis were evident (Figure-3e). Microscopically, all cattle had fibrinosuppurative broncho- or interstitial bronchopneumonia affecting much of the cranioventral aspect of the lung.

**Figure-4:**
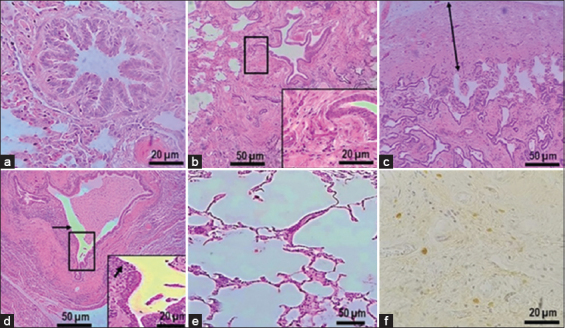
Lung histopathological lesions in chronic infection. (a) Normal alveoli and bronchiole. (b) Alveolar thickening with interstitial fibrosis. (c) Pleural fibrosis. (d) Bronchial dilatation. (e) Emphysema. (f) *Klebsiella pneumoniae* in interstitial lung.

In contrast, characteristics of chronic pneumonia such as thickened alveolar walls by interstitial tissue fibrosis (Figure-4b), pleural fibrosis (Figure-4c), and lymphocytes and macrophages infiltration, as well as bronchiole dilatation (Figure-4d) and emphysema were observed in several samples (Figure-4e). Destruction of epithelium appears in bronchitis compared to normal tissue (Figure-4a).

### IHC

hvKp antigens were present in the bronchiolar and alveolar lung epithelia, interstitium, and pleura. Specific immunoperoxidase labeling associated with the hvKp antigen was most frequently found in the cytoplasm of bronchiolar epithelial cells, neutrophils, macrophages, cytoplasm of the pneumocytes type II. In areas with encapsulated bacteria, *K. pneumoniae* antigen was rarely detected inside inflammatory cells. On the other hand, the cytoplasm of macrophages containing phagocytized debris of *K. pneumoniae* was observed in the area where bacteria were partially disrupted or lacking peripheral halos. In lungs with acute lesions, the distribution of *K. pneumoniae* antigens was mainly in the bronchiolar, vascular, and alveolar areas (Figure-3f), in contrast to largely diffuse infiltrates in the fibrotic areas of lungs with chronic lesions (Figure-4f).

## Discussion

Aceh cattle are indigenous Indonesian beef cattle that are raised with traditional to semi-intensive husbandry practices. However, rural farming with traditional methods is often faced with disease problems, especially respiratory diseases. Respiratory disease was frequently reported and often associated with pasteurellosis based on serological testing, although *Pasteurella multocida* as the actual causative agent was predominantly negative by PCR testing (personal communication).

In this study, we report the isolation of *K. pneumoniae* in all 15 pneumonic lungs, which is confirmed by tracing the *rpoB* gene. All isolates can be categorized as hvKp based on the string formation in blood agar. The *rpoB* gene that encodes the beta subunit of the bacterial RNA polymerase has been effectively used to identify *K. pneumoniae* [22], and it is more discriminative than the 16S rRNA gene, allowing identification at the species or even subspecies level of *K. pneumoniae* [24].

*K. pneumoniae* is an opportunistic pathogen predominantly affecting immunocompromised or elderly patients. Although *K. pneumoniae* is primarily known as the cause of environment-derived bovine mastitis, upper respiratory tract infection of dairy cattle with hvKp has been reported [1]. The presence of *K. pneumoniae* in pneumonic lungs has been reported as the second most prevalent after *Staphylococcus aureus* in mixed bacterial respiratory infection in cattle [20]. The incidence of hvKp in Aceh cattle lungs is presumably due to inhalation of pathogens from the upper respiratory tract.

Airway invasion was influenced by various host factors and the capability of *K*. *pneumoniae* pathotype to inhibit host defense responses by immune evasion strategies. hvKp employs many strategies to survive and protect itself from the host immune response [25]. *K. pneumoniae* has been associated with two forms of pneumonia in humans, acute and chronic infection. The acute form usually develops quickly and lasts less than 2 weeks, manifesting with symptoms such as high fever, dry cough, chest pain, and dyspnea [26]. In contrast, the chronic infection develops slowly, similar to other chronic lung infections, and frequently exhibited as a chronic productive cough [27]. Our finding suggested the involvement of hvKp in acute and chronic respiratory infection in Aceh cattle.

Acute pneumonia was marked by hyperemia (intense capillary congestion), polymorphonuclear leukocyte infiltrates, edema, alveolar hemorrhage, bronchiolar hyperplasia, and purulent exudates within the bronchus and bronchioles. In the early stage of the respiratory infection, a bacterial pathogen has to escape the first mechanical defense of mucociliary clearance in the bronchus [28,29]. Loss of cilia and ciliated cells in small bronchi and bronchioles has been shown to be responsible for the host defense failure against hvKp colonization through biofilm formation, and allows bacteria to access the lungs [25]. Infection of hvKp in the airways triggers hyperplasia of bronchiole cells, neutrophils infiltration, and purulent exudate production, leading to the obstruction of the bronchi and bronchioles. The airway constriction and obstruction trap the air in the lung periphery and eventually give rise to atelectasis. Simultaneously, cell death and LPS activation during bacterial infection will induce prostaglandins (PGE_2_) synthesis. PGE_2_-mediated increase in microvascular permeability and vasodilation causes increased blood flow (hyperemia) and facilitates the recruitment of inflammatory cells [30]. Neutrophils, macrophages, and mast cells release pro-inflammatory mediators such as tumor necrosis factor-alpha (TNF-α), interleukin 6, and interferon-gamma to induce the expression of adhesion molecules in the endothelium and promote neutrophils attachment to the vascular endothelium [31]. Furthermore, pro-inflammatory mediators will increase vascular permeability and immobilization of circulating neutrophils to the site of infection [32]. Subsequently, this vascular permeability causes plasma protein extravasation, causing edema. In addition to vascular permeability, pulmonary hemorrhage and edema can be caused by lung vascular injury [33].

Colonization of hvKp through biofilm formation facilitates evasion of the host defense system and resistance to antimicrobial agents, which most likely leads to chronic infection [34]. Our study suggests the presence of chronic hvKp lung infection in Aceh cattle as characterized by moderate thickening of alveolar walls, extensive interstitial fibrosis, infiltration of lymphocytes and macrophages, emphysema, and bronchiole dilatation. The previous study reported that *K. pneumoniae* invasion of sheep lungs causes hyperplasia of pneumocytes type II and infiltration of inflammatory cells such as lymphocytes and macrophages to the infection area, causing alveolar thickening [35]. In the case of chronic inflammation, alveolar tissue injury can occur due to the damage of alveolar epithelial cells and basal membrane, endothelial cell disruption, and in severe cases, can cause damage of elastic fibers. Damage to endothelial cells will cause the leakage of protein-rich fluid into the alveolar space accompanied by myofibroblast migration from the interstitium, which results in the formation of immature collagenous tissue [36]. If inflammation persists, lung infection results in fibrotic remodeling and gives rise to a loss of tissue integrity, alveolar collapse, and irreversible fixed fibrosis, which can lead to total lung damage [37,38].

Histological examinations of the lungs of Aceh cattle showed pleural thickening marked with the presence of connective tissue, suggesting pleural fibrosis, which can be caused by severe inflammation that is common in chronic infections. The pleural injury will induce the activation of mesothelial cells, which together with inflammatory cells in the pleural space will release fibroblast growth factors such as transforming growth factor-beta (TGF-ß), Basic fibroblast growth factor, platelet-derived growth factor, and connective tissue growth factor. In addition, pleural injury induces induction of the coagulation pathway and inhibition of fibrinolysis which mediated pleural fibrosis [39]. Pleural thickening characterized by marked expansion of fibrous tissue was demonstrated by *Klebsiella* spp. infection in horses [40].

Emphysema was evident in this study and was mostly combined with fibrosis. These could be attributable to constant and severe inflammation, which causes airflow obstruction, tissue destruction, extensive tissue structural alteration, and overexpression of immune mediators, such as TNF-α and TGF-β. Infiltration of inflammatory cells into the alveolar walls triggers alveoli destruction by neutrophil elastase and macrophage proteolytic enzymes, resulting in the enlargement of air spaces due to the degradation of the extracellular matrix, specifically collagen and elastin, in the alveoli [41,42]. Immunohistochemical staining demonstrated that the distribution of *Klebsiella* antigens was consistent with histopathological findings. In acute infection, hvKp antigens were detected in the alveolar, vascular, and bronchiolar areas, while in chronic infection, antigens were predominantly present in areas of active fibrosis. Furthermore, macrophages were rarely present in the areas having encapsulated bacteria, in contrast to areas with non-capsulated or disrupted bacteria, which were surrounded by macrophages containing antigens. Similar findings in sea lions were also reported [16], substantiating the importance of *K. pneumoniae* capsular antigen in escaping host immune responses, such that they can invade the lower respiratory system. Thus, the observations of the histopathological and immunohistochemical findings in the lungs of Aceh cattle suggest the involvement of hvKp presence in acute (9/15; 60%) and chronic (6/15; 40%) infections; however, the role of other respiratory pathogens such as *Mycoplasma*, *Mannheimia haemolytica*, and *P. multocida* has remained to be established.

## Conclusion

hvkp can be detected in the lungs of Aceh cattle representing acute and chronic infections. Acute infection was characterized by alveolar hyperemia and hemorrhage, neutrophils infiltration, and atelectasis, while the chronic infection was characterized by thickened alveolar walls, interstitial and pleural fibrosis, emphysema, and bronchiole dilatation. The distribution of *Klebsiella* antigens in lung tissue was consistent with histopathological findings. In view of all the findings, further investigations are necessary to determine infection of *K. pneumoniae*, alone or together with other potential pathogens in disease pathogenesis and progression.

## Authors’ Contributions

DD executed the work (sample collection and examination, data analysis, and writing manuscript). SS participated in the conception and microbiological study design, data analysis and interpretation, and drafting the manuscript. DRA analyzed and interpreted the pathological data and revised the manuscript draft. EH conceptualized, designed, analyzed, and interpreted the pathological data. All authors read and approved the final manuscript.
